# A German version of the Caregiver Skills scale for caregivers of patients with anorexia nervosa

**DOI:** 10.1002/erv.2817

**Published:** 2020-12-17

**Authors:** Michael Zeiler, Julia Philipp, Stefanie Truttmann, Tanja Wittek, Claudia Franta, Hartmut Imgart, Annika Zanko, Ellen Auer‐Welsbach, Leonie Kahlenberg, Gudrun Wagner, Andreas Karwautz

**Affiliations:** ^1^ Department for Child and Adolescent Psychiatry Eating Disorders Unit Medical University of Vienna Vienna Austria; ^2^ Parkland Clinic Clinic for Psychosomatic Medicine and Psychotherapy Bad Wildungen Germany; ^3^ Department for Neurology and Psychiatry of Children and Adolescents Klagenfurt am Wörthersee Austria

**Keywords:** anorexia nervosa, caregivers, CASK, eating disorders, skills, validity

## Abstract

**Objective:**

To investigate acceptance, reliability, convergent validity, factor structure and sensitivity to change of a German translation of the Caregiver Skills (CASK) scale measuring skills related to caring for patients with eating disorders.

**Methods:**

Two hundred and thirty‐three parents (76% female) of adolescent patients (mean age 15.1) with anorexia nervosa (AN) completed the 27 items of the CASK. We calculated item/scale characteristics, internal consistencies and bivariate correlations with other measures of caregiving burden. We evaluated goodness‐of‐fit of the 6‐factor model using confirmatory factors analysis and explored the sensitivity to change following two skills‐based trainings.

**Results:**

The fit of the 6‐factor model was acceptable (Root Mean Square Error of Approximation: 0.077, Standard Root Mean Square Residual: 0.080). Cronbach's alpha was excellent for the total (.94) and acceptable for all subscales (0.73–0.85). The total CASK score was 68.04 (max. 100) showing relatively high self‐rated caregiver skills. Non‐completion rates of most items were low (<3%) indicating high acceptance. Convergent validity was found with measures of psychological distress, depression, anxiety and expressed emotion. The total score significantly increased following an 8‐week workshop/online skills training (*d* = 0.70) and a 2‐day multi‐family intervention (*d* = 0.47).

**Discussion:**

The German CASK version is a useful instrument to assess caregiver skills in parents of patients with AN and to evaluate outcomes of skills‐based trainings.

## Key points

- This study provides the first translation and evaluation of a German version of the Caregiver's skills scale (CASK)
- The CASK showed high acceptance across caregivers, good internal consistencies of the total and subscales and acceptable fit of the 6‐factor model
- Caregiver's skills as measured with the CASK improved with medium‐to‐high effect sizes following skill‐based training/multi‐family therapy based on the cognitive interpersonal maintenance model of eating disorders

## 1 INTRODUCTION

Caregivers of adolescent patients with anorexia nervosa (AN) play a key role in eating disorder (ED) treatment and, if adequately trained, may facilitate recovery from this illness (Treasure & Nazar, 2016). They are often facing difficulties and uncertainties in their caregiving role (e.g. uncertainties regarding factors relevant to the causes of EDs, interpersonal difficulties, arguments during mealtime), which may lead to inappropriate cognitive beliefs (e.g. self‐blame) and maladaptive behaviour such as high expressed emotion. In further consequence, this may contribute to the maintenance of the adolescent's AN and increases caregivers' own psychopathology (Haigh & Treasure, 2003; Rhind et al., 2016; Treasure et al., 2008; Treasure & Nazar, 2016). Previous research has highlighted the benefits of skills‐based trainings for caregivers of patients with EDs and found a reduction of caregiver distress, burden and expressed emotion (Hibbs, Rhind, Leppanen, & Treasure, 2015b; Philipp, Truttmann, et al., 2020; Truttmann et al., 2020), as well as improvements of ED symptomatology in patients (Hibbs et al., 2015a; Philipp, Franta, et al., 2020).

So far, different instruments were used to assess the burden and needs of caregivers of patients with EDs. Apart from general measures of psychopathology (including depression, anxiety and stress) which were not validated in the field of EDs, instruments for specific use in caregivers of ED patients were developed; some of them are available in various languages. For example, the ‘Carers’ Needs Assessment Measure’ (Haigh & Treasure, 2003) was developed to assess caregivers' needs and unmet needs in caring for someone with an ED, the ‘Eating Disorder Symptom Impact Scale’ (Sepulveda et al., 2008) measures the caregivers' burden related to the ED in the family and the ‘Accommodation and Enabling Scale for Eating Disorders’ (Sepulveda et al., 2009) measures the extent to which relatives adapt their lives to the ED situation in the family. Available instruments specifically addressing caregiver skills regarding how to deal with the ED in the family are scarce and focus on specific aspects, like self‐efficacy (Rhodes et al., 2005), only. Moreover, caregiver questionnaires developed in the context of other disorders (e.g. dementia, psychosis) are not suitable for caregivers of ED patients due to their specific challenges (e.g. difficulties during mealtimes, with weight control). Thus, the development of new instruments assessing caregiver skills in the field of ED is needed.

Hibbs et al.  (2015c) have developed a new questionnaire [Caregiver Skills scale (CASK)] to adequately assess caregiver skills in caregivers of patients with AN and evaluated outcomes of skills‐based trainings. Items included in this questionnaire comprise core skills and values taught in caregiver interventions in line with the cognitive interpersonal maintenance model of EDs (Goddard et al., 2011; Schmidt & Treasure, 2006). The good fit between item content and content conveyed in this type of intervention can be regarded as one of the key strengths of this questionnaire. The skills assessed in the CASK include communication about the illness in a compassionate way based on Motivational Interviewing (Miller & Rollnick, 2002) and conveying hope and confidence, keeping a focus on the bigger picture rather than focusing on details of the ED, accepting the illness rather than running into self‐blame or blaming others and caring for self and other family members not affected by the illness. Previous studies found that caregiver skills as measured with CASK significantly improved in caregivers participating in workshop or guided self‐help interventions with medium‐sized effects (Adamson et al., 2019; Hodsoll et al., 2017; Jenkins et al., 2018). An exploratory factor analysis revealed six factors with internal consistencies of all subscales being in the high to acceptable range (Hibbs et al., 2015c). The authors also reported reasonable convergent validity of the CASK with other measures of caregiver distress, anxiety and expressed emotion. Recently, the 6‐factor structure of the original version was confirmed in a Spanish translation of the CASK with no differences in the CASK scores between mothers and fathers of patients with any kind of EDs (Vintró‐Alcaraz et al., 2018).

Caregiver skills trainings building on the cognitive interpersonal maintenance model of EDs are increasingly developed and implemented in German‐speaking countries, such as Austria and Germany (Franta et al., 2018; Spencer et al., 2019). However, instruments appropriately evaluating effects of these interventions in the German‐speaking population are scarce, and none is focussing on skills specifically relevant for caregivers of ED patients. Thus, the aim of the present study was to evaluate a German version of the CASK in a sample of caregivers of adolescent patients with AN. We used confirmatory factor analysis (CFA) to test the factorial structure proposed by the authors of the original English version and investigated item and scale characteristics (i.e. internal consistencies), as well as the convergent validity with a variety of measures assessing caregiver's burden and distress. Furthermore, we explored whether the CASK is sensitive to change following two different types of caregiver interventions which is an important premise to evaluate the efficacy of such interventions. We hypothesised that the 6‐factor structure of the German CASK version is confirmed, and the level of caregiver skills is significantly negatively associated with measures of caregiver's burden. Moreover, we expected that the level of caregiver skills as measured with the CASK significantly increases after having participated in a specialised caregiver intervention.

## 2 METHODS

### 2.1 Sample

Data used for the present study were obtained in the course of the Supporting Carers of Children and Adolescents with Eating Disorders in Austria (SUCCEAT) project. Inclusion criteria were having a child in current treatment due to AN, willingness to participate in a caregiver intervention and being fluent in German language. We excluded from this study caregivers who were not fluent in German, caregivers with severe psychiatric disorders (e.g. psychosis) and caregivers of patients with severe comorbidity. Of those eligible (*N* = 288), the response rate was 81.2% (*N* = 233). The sample includes *N* = 149 caregivers of adolescent patients with AN who participated in a clinical trial evaluating the efficacy and feasibility of the SUCCEAT workshop versus online caregiver intervention compared to an active comparison group (Franta et al., 2018) and *N* = 84 caregivers who participated in an ongoing follow‐up project evaluating the implementation of the SUCCEAT intervention in routine care. These figures are totalling up to *N* = 233 used for the present study. We used the baseline assessments to analyse the validity and item/scale characteristics of the CASK questionnaire. SUCCEAT participants were recruited from the Department of Child and Adolescent Psychiatry at the Medical University of Vienna. Caregivers of the comparison group were recruited from the Clinic for Psychosomatic Medicine and Psychotherapy, Parkland Clinic, Bad Wildungen and the Department for Neurology and Psychiatry of Children and Adolescents, Klagenfurt.

Of the 233 participants with a mean age of 47.73 years (SD = 5.03), 177 (76.0%) were mothers, 54 (23.2%) were fathers and 2 (0.9%) were stepfathers. Most caregivers had a university degree (45.9%) followed by A level degree (26.4%) and below A level degree (27.7%). Most caregivers were married or lived in a partnership (78.6%) while 15.7% were divorced or widowed and 5.7% were single. Information of caregivers from 206 adolescent patients with AN (primarily restrictive type) with a mean age of 15.10 (SD = 1.83) were obtained. ED duration in patients was 15.46 months (SD = 11.00) on average. A total of 54.9% of the adolescent patients received inpatient and 45.1% outpatient treatment. Sample characteristics of caregivers and patients divided by subgroups are shown in Table 1.

**TABLE 1:** Sample characteristics

	Total sample	SUCCEAT main trial (Franta et al., 2018)		SUCCEAT follow‐up trial
		SUCCEAT group	Control group	
*N* caregivers	233	100	49	84^a^
Mothers (*N*, %)	177 (76.0%)	86 (86.0%)	37 (75.5%)	54 (64.3%)
Fathers (*N*, %)	54 (23.2%)	14 (14.0%)	12 (24.5%)	28 (33.3%)
Other caregivers (*N*, %)	2 (0.9%)	0 (0.0%)	0 (0.0%)	2 (2.4%)
Age (mean, SD)	47.73 (5.03)	47.18 (4.88)	47.27 (4.48)	48.67 (5.43)
Education (*N*, %)				
University degree	106 (45.9%)	53 (53.0%)	13 (27.7%)	40 (47.6%)
A level degree	61 (26.4%)	22 (22.0%)	15 (31.9%)	24 (28.6%)
<A level degree	64 (27.7%)	25 (25.0%)	19 (40.4%)	20 (23.8%)
Unknown	2		2	
Marital status (*N*, %)				
Single	13 (5.7%)	3 (3.1%)	2 (4.3%)	8 (9.5%)
Married or in partnership	180 (78.6%)	78 (79.6%)	33 (70.2%)	69 (82.1%)
Divorced or widowed	36 (15.7%)	17 (17.3%)	12 (25.5%)	7 (8.3%)
Unknown	4	2	2	
*N* patients	206	100	49	57^a^
Patients' sex (*N*, %)				
Females	198 (96.1%)	93 (93.0%)	49 (100%)	56 (98.2%)
Males	8 (3.9%)	7 (7.0%)	0 (0.0%)	1 (1.8%)
Patients' age (mean, SD)	15.10 (1.83)	14.89 (1.86)	15.43 (1.08)	15.18 (2.22)
ED duration in months (mean, SD)	15.46 (11.00)	13.44 (12.98)	23.77 (12.93)	12.27 (12.75)
Type of treatment (*N*, %)				
Inpatient	113 (54.9%)	48 (48.0%)	40 (81.6%)	25 (43.9%)
Outpatient	93 (45.1%)	52 (52.0%)	9 (18.4%)	32 (56.1%)

^a^ In the SUCCEAT follow‐up trial both parents were included in the study if possible leading to a sample of 84 caregivers who cared for a total of 57 adolescent patients.

To explore the CASK's sensitivity to change, a subsample of 118 caregivers (those who participated in the SUCCEAT main trial and provided baseline and 3‐months post intervention assessments) was used. The manualised SUCCEAT intervention (subsample of 94 caregivers), fully described in Franta et al. (2018), is based on the cognitive interpersonal maintenance model of EDs (Schmidt & Treasure, 2006) and comprised eight weekly workshop or guided online sessions to improve caregiver skills. The comparison group (subsample of 24 caregivers) received multi‐family therapy via 2‐day workshops (Imgart & Plassmann, 2020) which also includes elements of the cognitive interpersonal maintenance model of EDs.

### 2.2 Instruments

CASK (original English version: Hibbs et al., 2015c): The CASK comprises 27 items assessing a variety of caregiver skills around communication, compassionate behaviour, accepting attitude and self‐care. Items are rated on a visual analogue scale with anchors 0 and 100 with higher values representing higher self‐assessed skills levels. The authors of the original English version proposed six factors including the ability to be positive about changes (‘Bigger Picture’, 7 items), to take time for oneself and other family members (‘Self Care’, 4 items), to avoid repetitive nagging conversations (‘Biting‐Your‐Tongue’, 3 items), to accept and manage negative emotions (‘Insight and Acceptance’, 3 items), to discuss and manage feelings (‘Emotional Intelligence’, 5 items) and to side step conflict and be calm and understanding (‘Frustration Tolerance’, 5 items). Item assignments to these scales are shown in Table 2. Additionally, a total score is calculated by aggregating all item ratings. Cronbach's alpha of the English version was excellent for the total score (0.92) and acceptable for all subscales (0.71–0.85). The instruction and items of the CASK were translated into German and back‐translated into English by two bilingual psychologists experienced in the field of EDs. Inconsistencies were discussed and resolved by consensus. Due to ambiguity regarding the meaning of item #25, we decided to slightly reframe the original item *‘Accept that the one cause or trigger for the eating disorder may not be the solution to recovery’* to ‘*Accept that there is no just one cause/one trigger for the development of an eating disorder*’. Furthermore, we added the option to not respond to an item by checking ‘*I do not understand the point of this question’*. The German version of the CASK is provided in Data S1. We calculated mean scores for the total scale and the subscales. Thus, these scores range between 0 (low skills) and 100 (excellent skills).

**TABLE 2:** Results of the confirmatory factor analysis (6‐factor model) and item/scale characteristics

Factors and items	CFA factor loadings				Item/scale characteristics			
	*B*	SE	*p*	*β*	Mean	SD	Median	Missing (%)
F1. Bigger picture (*α* = 0.846)					73.80	15.40	75	
09. Reassured by even small improvement	14.048	1.642	<0.001	0.654	78.17	21.46	80	2.6
10. Keep hope that X will recover	12.057	1.355	<0.001	0.630	83.94	19.18	90	0.4
17. Praise change or attempts	14.399	1.135	<0.001	0.687	72.85	20.97	80	1.3
20. Keep your eye on X's progress	15.180	1.269	<0.001	0.757	73.44	20.15	80	2.1
21. Resist in relying solely on weight	15.029	1.302	<0.001	0.665	74.74	22.69	80	2.6
22. Separate X as a person	14.740	1.417	<0.001	0.597	69.31	24.76	70	3.0
23. Reflect and understand	13.227	1.399	<0.001	0.616	63.06	21.62	70	9.4
F2. Self‐care (*α* = 0.781)					61.21	19.00	63	
01. Keep doing things that you enjoy	18.146	1.808	<0.001	0.694	56.83	26.15	60	1.7
07. Take some time for yourself	19.656	1.780	<0.001	0.750	51.21	26.28	50	0.4
11. Step back and trust	13.000	1.883	<0.001	0.584	65.02	22.30	70	0.4
26. Find time to spend with family	17.054	1.167	<0.001	0.757	72.11	2.56	80	1.3
F3. Biting‐your‐tongue (*α* = 0.833)					59.20	20.89	60	
16. Control urge enquiring checking	21.776	1.291	<0.001	0.875	60.80	24.91	60	0.9
18. Resist constantly remind and ask	20.091	1.180	<0.001	0.857	60.41	23.52	60	1.7
19. Avoid getting in conversations	16.143	1.472	<0.001	0.672	56.39	24.09	50	1.3
F4. Insight and acceptance (*α* = 0.728)					71.88	19.59	77	
24. Accept that ED is not your fault	19.342	2.093	<0.001	0.710	65.87	27.24	70	1.7
25. Insight there is no one cause	12.610	1.994	<0.001	0.601	84.32	21.02	90	2.1
27. Manage your anxiety levels	17.816	1.701	<0.001	0.739	65.20	24.16	70	2.1
F5. Emotional Intelligence (*α* = 0.780)					68.99	18.08	70	
02. Discuss and explain feelings	15.123	2.056	<0.001	0.523	68.25	28.99	80	1.7
03. Discuss the ED openly with family	12.863	1.999	<0.001	0.495	75.41	26.05	80	1.3
08. Talk and listen with X emotions	15.417	1.660	<0.001	0.610	72.06	25.32	80	0.9
12. Agree boundaries‐plans	16.325	1.447	<0.001	0.727	65.56	22.52	70	0.9
13. Uphold boundaries/rules	15.917	1.290	<0.001	0.740	63.93	21.64	70	2.1
F6. Frustration tolerance (*α* = 0.818)					67.94	16.28	70	
04. Be understanding towards X	13.664	1.225	<0.001	0.727	71.13	18.81	70	0.9
05. Avoid drawn into arguments	16.936	1.153	<0.001	0.764	67.23	22.19	70	1.7
06. Be calm with difficult ED behaviour	16.691	1.202	<0.001	0.776	62.21	21.52	70	0.9
14. Control the urge to argue	15.148	1.508	<0.001	0.646	58.10	23.54	60	6.4
15. Pleasant verbal interactions	11.627	1.600	<0.001	0.554	80.15	21.02	90	0.4

*Note:* Model fit: RMSEA = 0.077; SRMR = 0.080; CFI = 0.826.

We further selected measures of general psychological distress, depression, anxiety, eating disorder related difficulties and expressed emotion as relevant measures for evaluating the convergent validity of the CASK as previous studies have reported that higher skills in caring for a person with an eating disorder is associated with lower levels caregiver's distress and psychopathology (Goodier et al., 2014; Sepúlveda et al., 2012). Furthermore, the concept of expressed emotion is directly linked to caregiving skills (Kyriacou et al., 2008a).

General Health Questionnaire (GHQ; Goldberg et al., 1997; Linden et al., 1996): The GHQ comprises 12 items rated on a four‐point scale and is a measure of general psychological distress. Item ratings are dichotomised and summed up to a total score ranging from 0 to 12 with higher scores indicating higher levels of psychological distress. The internal consistency of the German 12‐item version was high (Cronbach's *α*: 0.91; Schmitz et al., 1999; our study sample: *α* = 0.89).

Beck Depression Inventory (BDI‐II; Hautzinger et al., 2006): The 21 items of the BDI‐II assess core symptoms of depression. Items are rated on a four‐point scale and are aggregated to a total score with higher scores indicating higher levels of depression. In a German sample of healthy adults, a Cronbach's *α* of 0.90 was reported (Hautzinger et al., 2006); in our sample, Cronbach's *α* was 0.88.

State/Trait Anxiety Inventory (STAI; Laux et al., 1981): State and trait anxiety is measured by 20 items each rated on a four‐point scale. Higher scores indicate higher state/trait anxiety levels. Internal consistencies were high for the state and trait anxiety scale (*α* = 0.90 for both scales; Laux et al., 1981). In our sample, Cronbach's *α* was 0.94 for the state anxiety and 0.93 for trait anxiety scale.

Eating Disorder Symptom Impact Scale (EDSIS; Sepulveda et al., 2008): The EDSIS is a measure of caregiving difficulties for families of people with an ED and comprises difficulties in specific areas such as arguments during meal time, dysregulated behaviour, feelings of guilt and social isolation. A total of 24 items rated on a five‐point scale are summed up to a total score with higher scores indicating larger difficulties. For the purpose of this study, we used the total score only. The German version of the EDSIS was developed by our research team using the translation‐back translation principle and is currently being validated. The Cronbach's *α* for the total scale (English version) was high (*α* = 0.90; Sepulveda et al., 2008). In our sample, Cronbach's *α* was 0.88.

Family Questionnaire (FQ; Wiedemann et al., 2002): The FQ is a measure of expressed emotion in caregivers of ED patients including two aspects, emotional over‐involvement and criticism. The 20 items are rated on a four‐point scale and summed up to an emotional over‐involvement and criticism score with higher scores indicating higher levels of expressed emotion. Internal consistencies in the German version were high (*α* = 0.92) for the criticism scale and acceptable (*α* = 0.79) for the emotional over‐involvement scale (Wiedemann et al., 2002). In the present sample, Cronbach's alphas were 0.87 and 0.80, respectively.

### 2.3 Procedure

Written informed consent was provided from all caregivers involved in this study. Ethical approval was obtained from the Ethics Committee of the Medical University of Vienna (EK 1840/2013). The data collection in this study adhered to the ethical standards laid down in the 1964 Declaration of Helsinki and its later amendments. Details on the study procedures and interventions to analyse the CASK's sensitivity to change are published elsewhere (Franta et al., 2018).

### 2.4 Statistical analyses

We performed a CFA using the package ‘lavaan’ in *R* (Rosseel, 2012) to test the 6‐factor model of the CASK proposed by the authors of the original English version (Hibbs et al., 2015c). Cases with missing CASK data were included using full information maximum likelihood estimation. We used the Huber‐White robust maximum likelihood estimator for calculating standard errors accounting for small deviation from a normal distribution. We selected the Root Mean Square Error of Approximation (RMSEA) as the primary measure for goodness‐of‐fit but also report the Comparative Fit Index (CFI) as this fit measure is known to be less affected by sample size and the Standard Root Mean Square Residual (SRMR) as this measure is less affected by the model complexity. It has been suggested that RMSEA values below 0.05 can be regarded as good, values between 0.05 and 0.08 as acceptable, values between 0.08 and 0.10 as marginal and values above 0.10 as poor (Fabrigar et al., 1999). CFI values above 0.9 and SRMR values below 0.1 indicate acceptable model fits (Bentler, 1990; Cangur & Ercan, 2015). Unstandardised and standardised factor loadings were calculated. Furthermore, we compared goodness‐of‐fit between the 6‐factor and a one‐factor model (all items loading on one global factor).

In order to investigate internal consistencies, we calculated Cronbach's alpha and corrected item‐scale correlations for all CASK scales. We used the Feld test to analyse differences in the internal consistencies between female and male caregivers (Feldt, 1969).

We calculated descriptive statistics to explore the number and percentage of missing items for the total CASK questionnaire and on item‐level to provide the mean, median and standard deviations of item ratings and scales. Deviations from a normal distribution were analysed using the Kolmogorov–Smirnov test and using graphical explorations (histogram, boxplots). We used a *t*‐test to analyse differences between female and male caregivers and between caregivers of inpatients and outpatients regarding the CASK total score and subscales. For assessing the convergent validity, Pearson correlation coefficients were calculated between the CASK scores and the GHQ, FQ, BDI, STAI and EDSIS total scores. According to Cohen (1988), *r* = 0.1, *r* = 0.3 and *r* = 0.5 were interpreted as small, medium and large effects, respectively.

The CASK's sensitivity to change was investigated using dependent *t*‐tests, calculating differences between the baseline and post‐intervention scores. We performed this analysis separately for caregivers who participated in the SUCCEAT intervention and for those who received multi‐family therapy. Effect sizes are provided in terms of Cohen's *d*; values of 0.2, 0.5 and 0.8 were interpreted as small, medium and large effects, respectively (Cohen, 1988). This study aimed to give rough estimation on whether the CASK is sensitive to change following a caregivers' skills training. A detailed analysis on the effectiveness of the SUCCEAT intervention including between group differences is provided by Truttmann et al. (2020).

## 3 RESULTS

### 3.1 Factorial validity

A CFA was performed to assess goodness‐of‐fit of the 6‐factor model of the CASK. Among the total sample, goodness‐of‐fit was within the acceptable range regarding the RMSEA [0.077 (90%CI: 0.070; 0.084)] and the SRMR (0.080) index, but marginal regarding the CFI (0.826). All standardised factor loadings of items assigned to the respective subscale were statistically significant (all *p*‐values < 0.001) and ranged between 0.495 and 0.875 (mean: 0.682, SD: 0.093). Unstandardised and standardised factor loadings are shown in Table 2. The CFA path diagram including factor loadings and correlations between the latent factors are provided in Figure S1. Inspecting the model revealed considerable residual correlations for seven items (#1, #2, #3, #10, #11, #15, #19); thus, we re‐specified the original model by including item covariances of the aforementioned items in the model. Doing so, goodness‐of‐fit improved [RMSEA: 0.051 (90%CI: 0.042; 0.065), SRMR: 0.063, CFI: 0.937]. Factor loadings of this re‐specified model are provided in Table S3. Goodness‐of‐fit of a one‐factor model was worst [RMSEA: 0.094 (95%CI: 0.088; 0.100), SRMR: 0.078; CFI: 0.730] (factor loadings not displayed).

### 3.2 Internal consistencies

The internal consistency of the CASK total scale was excellent (Cronbach's alpha: 0.936) with corrected item‐scale correlations ranging from 0.432 to 0.721. For the subscales, the Cronbach's alpha were all in the acceptable to good range: ‘Bigger Picture’: *α* = 0.846, ‘Self‐Care’: *α* = 0.781, ‘Biting Tongue’: *α* = 0.833, ‘Insight and Acceptance: *α* = 0.728, ‘Emotional Intelligence’: *α* = 0.780, ‘Frustration Tolerance’: *α* = 0.818. The corrected item‐scale correlations ranged between 0.417 and 0.755 across all subscales. Cronbach's alphas for the CASK scales divided by caregiver's sex are provided in Table S2. Using Bonferroni‐corrected significance levels, the Cronbach's alpha for the ‘Biting Tongue’ subscale was significantly higher in mothers compared to fathers (Feldt test *W* = 0.577, *p* = 0.004). Regarding the other CASK scales, there was no significant differences between mothers and fathers.

### 3.3 Item and scale characteristics

The total number of missing CASK items ranged from 0 to 16 with 74.7% having completed all items. Of participants with missing items, the mean number of missing items was 2.07 (SD: 2.71). The percentage of missing data was between 0% and 3% for all CASK items except for item #14 and item #23 where non‐completion rates were 6.4% and 9.4%. Whereas the CASK total mean score was compatible with normal distribution (Kolmogorov–Smirnov‐*Z* = 0.045, *p* ≥ 0.200), all subscales showed small but statistically significant deviations from normal distribution (negatively skewed) with *p*‐values between 0.047 and 0.001. We also checked the distribution graphically (histogram, boxplots) and observed only minimal deviations from a normal distribution; thus parametric statistical tests were used in the subsequent analyses which should be robust for the given sample size (*N* > 200). The CASK total mean score was 68.04 (95%CI: 66.19; 69.90) (SD: 14.25) with scores ranging from 21.85 to 100. Female caregivers showed statistically significant lower total scores [mean: 66.83 (95%CI: 64.74; 68.92) SD: 14.03] compared to male caregivers [mean: 72.01 (95%CI: 68.08; 75.94), SD: 14.40; *t* = 2.338, *p* = 0.019]. The effect size for this difference was small (Cohen's *d* = 0.37). Regarding the subscales, significantly higher scores in females were observed for the ‘Self‐Care’ (*p* = 0.012), ‘Biting Tongue’ (*p* = 0.010) and ‘Acceptance’ (*p* < 0.001) subscales. There was no statistically significant difference in the CASK total and sub‐scores between caregivers of inpatient and outpatient adolescents (*t* = 0.079, *p* = 0.937). The item and scale characteristics including mean, standard deviation, median and percentage of missing data are shown in Table 2. The CASK percentiles for the total and sub‐scales (for the total sample and divided by caregiver's sex) are provided in Table S1.

### 3.4 Convergent validity

Bivariate correlations of the CASK scales with the GHQ, FQ, BDI, STAI and EDSIS are shown in Table 3. Negative correlations were found between the CASK scales and all other considered outcome variables (all *p*‐values < 0.001), indicating that higher levels of caregiver skills were associated with lower levels of psychological distress, high expressed emotion, depression, anxiety and ED specific difficulties in the family. All correlation coefficients were in the medium‐to‐high range.

**TABLE 3:** Convergent validity of the CASK with other caregiver measures (Person correlation coefficients)

	1	2	3	4	5	6	7	8	9	10	11	12	13
1. CASK total	‐	‐	‐	‐	‐	‐	‐	‐	‐	‐	‐	‐	‐
2. CASK bigger picture	0.881	‐	‐	‐	‐	‐	‐	‐	‐	‐	‐	‐	‐
3. CASK self‐care	0.760	0.541	‐	‐	‐	‐	‐	‐	‐	‐	‐	‐	‐
4. CASK biting‐your‐Tongue	0.774	0.627	0.554	‐	‐	‐	‐	‐	‐	‐	‐	‐	‐
5. CASK insight and acceptance	0.729	0.563	0.567	0.556	‐	‐	‐	‐	‐	‐	‐	‐	‐
6. CASK emotional Intelligence	0.810	0.690	0.545	0.419	0.501	‐	‐	‐	‐	‐	‐	‐	‐
7. CASK frustration tolerance	0.830	0.687	0.512	0.663	0.457	0.622	‐	‐	‐	‐	‐	‐	‐
8. GHQ	−0.397	−0.243	−0.464	−0.330	−0.459	−0.274	−0.242	‐	‐	‐	‐	‐	‐
9. FQ: CC	−0.521	−0.434	−0.372	−0.425	−0.380	−0.412	−0.495	0.334	‐	‐	‐	‐	‐
10. FQ: EOI	−0.492	−0.358	−0.539	−0.401	−0.550	−0.344	−0.279	0.597	0.638	‐	‐	‐	‐
11. BDI	−0.494	−0.335	−0.491	−0.424	−0.572	−0.318	−0.327	0.681	0.325	0.579	‐	‐	‐
12. STAI‐state	−0.522	−0.356	−0.517	−0.442	−0.488	−0.355	−0.411	0.588	0.322	0.535	0.750	‐	‐
13. STAI‐trait	−0.515	−0.357	−0.496	−0.393	−0.574	−0.402	−0.316	0.555	0.330	0.494	0.755	0.728	‐
14. EDSIS	−0.460	−0.274	−0.526	−0.449	−0.503	−0.283	−0.296	0.559	0.587	0.708	0.522	0.504	0.486

*Note:* All correlation coefficients are significant on a significance level of 0.001.

Abbreviations: BDI, Beck Depression Inventory; CASK, Caregiver Skills Scale; EDSIS, Eating Disorder Symptom Impact Scale, FQ, Family Questionnaire (CC, Criticism; EOI, Emotional Over‐Involvement), GHQ, General Health Questionnaire; STAI, State/Trait Anxiety Inventory.

### 3.5 Sensitivity to change

Among caregivers who participated in the SUCCEAT intervention, the CASK total score significantly increased from baseline to the post‐intervention assessment (*t* = 6.570, *p* < 0.001) with an effect size of *d* = 0.70 [95%CI: 0.47; 0.94]. Furthermore, the subscale scores significantly improved (all *p*‐values < 0.01) with the highest effect size for the ‘Biting Tongue’ subscale (*d* = 0.89) and lowest effect size for the ‘Emotional Intelligence’ subscale (*d* = 0.31). Among caregivers who received multi‐family therapy, a statistically significant pre‐post improvement in total caregiver skills was observed as well (*t* = 2.270, *p* = 0.033); however, the effect size was lower compared to SUCCEAT participants (*d* = 0.47 [95%CI: 0.03; 0.92]). Regarding the subscales, statistically significant improvements were observed for the ‘Self‐Care’ (*p* = 0.019; *d* = 0.45) and ‘Biting Tongue’ (*p* = 0.023; *d* = 0.58) subscale while there was no statistically significant change in the other subscales.

## 4 DISCUSSION

This is the first study providing a German translation of the CASK scale and assessing its acceptance, psychometric properties, convergent validity, factor structure and sensitivity to change. Overall, we found a low number of missing items and acceptable to high internal consistencies of the total and sub‐scales of the German CASK version. Moreover, convergent validity was demonstrated with several measures of caregivers' psychological distress and expressed emotion. The CFA revealed acceptable model fit of the 6‐factor structure. We found that the CASK is sensitive to detect short‐term improvements in skill levels of caregivers who participated in an 8‐week skills training program (SUCCEAT) and—with lower effect sizes—also of caregivers receiving multi‐family therapy.

The German version of the CASK demonstrated satisfactory item and scale distributions. However, with a total mean score of 68.04 (of a maximum of 100), caregivers' self‐rated skills levels prior to the start of a specific intervention were in the upper range of all possible scores. In practical‐clinical terms, this score can be interpreted as a high skills level (Vintró‐Alcaraz et al., 2018). This average score was slightly higher than in a study in the United Kingdom (Hibbs et al., 2015c) and comparable with a sample of caregivers in Spain (Vintró‐Alcaraz et al., 2018). In contrast to other studies using the CASK (Salerno et al., 2016; Vintró‐Alcaraz et al., 2018), we found that male caregivers had slightly but significantly higher skills levels than female caregivers. Previous research in the ED field and other areas including autism, cerebral palsy and dementia indicates that female caregivers often show higher levels of stress and psychological burden than male caregivers (Gallicchio et al., 2002; Khanna et al., 2011; Kyriacou et al., 2008b; Penning & Wu, 2016), which has also been associated with the fact that females often spend more time caregiving than males (Byrne et al., 2010). Assuming that the self‐rated skills level is mediated by the level of perceived psychological distress, this may have resulted in the gender difference found in the present study. However, further research has to address this hypothesis.

The number and percentage of missing items can be regarded as a measure of questionnaire‐acceptance (Rick et al., 2012). In the present study, the percentage of item non‐completion was below 3% for most items indicating high acceptance and understanding. For two items (#14 ‘*Control the urge to argue against the eating disorder behaviours, even though you believe your argument to be logical.’* and #23 ‘*Reflect and understand the effect of your behaviour on N.N.’*), the percentage of non‐completion was higher (6% and 9%), which indicates that the understanding may benefit from rewording. Qualitative analysis, for example using the think‐aloud technique while completing the questionnaire (Nitsch et al., 2019; Willis & Artino, 2013), may provide further information on how item wordings can be improved.

The internal consistencies of the total and sub‐scales were all in the acceptable to high range and comparable to those of the original English CASK version (Hibbs et al., 2015c). Convergent validity of the CASK total score and the sub‐scores was demonstrated by observing medium‐to‐high bivariate negative correlations with different measures of caregivers' psychological distress, depression, anxiety, expressed emotion and difficulties in the family. This is consistent with Hibbs et al.  (2015c) who found that the CASK scores were significantly associated with depression and anxiety, general wellbeing, high expressed emotion and the accommodation and enabling scale for EDs. Improving caregiver skills (e.g. aspects of the relationships with the patients) have been directly linked to a decrease in the caregivers' levels of anxiety and depression (Sepúlveda et al., 2012). Furthermore, evidence from qualitative studies support that parental skills and coping strategies regarding the ED in the family are associated with psychological distress (Goodier et al., 2014), which also supports the findings of the present study.

In the CFA, the 6‐factor structure of the German version of the CASK yielded acceptable model fit regarding the RMSEA and SRMR index, but poor fit when considering the CFI. The reasons for the low CFI may lay in model complexity. Although we met the rule of thumb of a minimum required sample size of 200 (Barrett, 2007), our sample might have been still too small regarding the complexity of the model (6 factors with 27 items). However, the standardised factor loadings of all items are statistically significant and can be regarded as reasonable (44% of all factor loadings > 0.7, 37% > 0.6 and 19% ≥ 0.5). The model fit found in the present sample was worse than the model fit found in a sample who completed a Spanish translation of the CASK (Vintró‐Alcaraz et al., 2018). We explored whether the model fit increases when considering item residual covariances in the 6‐factor model and whether a one‐factor solution might fit better to the data. While the one‐factor model resulted in worse model fit, goodness‐of‐fit of the 6‐factor model improved significantly when allowing residual item covariances for seven items. The factor structure of the German CASK version should be further evaluated in future studies using different samples.

Finally, we aimed to explore that CASK's sensitivity to change following a skill‐based workshop/online program (SUCCEAT) which is based on the cognitive interpersonal maintenance model of EDs (Goddard et al., 2011; Schmidt & Treasure, 2006). In the short term (3 months after baseline assessment), the caregiver skills as measured with the CASK significantly increased with medium‐to‐high effect sizes for the total and most of the sub‐scales. This is congruent with UK studies that demonstrated that caregiver skills can be improved with similar interventions (Hodsoll et al., 2017; Jenkins et al., 2018). It is noteworthy that we found these effect sizes even though the self‐rated skills were already rather high at baseline which underlies the usefulness of the German version of the CASK to evaluate treatment effects in this type of intervention. Moreover, we explored whether the CASK was also useful to evaluate outcomes of multi‐family therapy. Short‐term effects were lower compared to the SUCCEAT intervention but still in the medium range (*d* = 0.47 for the total score), indicating the usefulness of the CASK for evaluation studies of multi‐family therapy in EDs as well. The improvements were statistically significant for the total and two subscales only; however, the low sample size in this subgroup must be considered when interpreting these results. The majority of topics of the multi‐family intervention were also based on principles of the cognitive interpersonal maintenance model of EDs and overlap with topics of the SUCCEAT intervention, including content around understanding factors relevant for the aetiology and course of EDs, improving family communications and reducing criticism (Imgart & Plassmann, 2020). In the present study, the duration of multi‐family therapy in terms of number of hours was comparable to the SUCCEAT intervention. However, the entire multi‐family intervention was delivered within two days whereby the sessions of the SUCCEAT intervention were delivered once a week over a duration of 8 weeks. This also may explain the differences in effects found in this study.

This study has the following limitations: Male caregivers (fathers) were underrepresented in this sample, which is a common phenomenon in studies with caregivers of ED patients (Hodsoll et al., 2017; Jenkins et al., 2018; Rhind et al., 2016). Thus, fitting the CFA model separately for sex and testing of measurement invariance of the factor structure of the CASK across female/male subgroups was not possible due to the low number of male caregivers who participated in this study. Future studies should put efforts in recruiting also a larger number of male caregivers to more elaborately investigate differences in caregiver skills between mothers and fathers of ED patients. Only caregivers of adolescent patients with AN were included. We cannot say whether the German version of the CASK is also useful and valid for caregivers of patients with bulimia nervosa or binge‐eating disorder. We did not exclude data from mother‐father dyads (*N* = 27) who are caregivers of the same patient. This was done because the CASK is designed to assess individual caregiver skills which are also likely to vary in mothers and fathers of the same patient. Indeed, there was no difference in the variance of CASK scores between data from families from whom one versus two caregivers participated in this study. Finally, one may argue that we included a heterogeneous sample of study participants recruited from different sites who differ in some sociodemographic characteristics (e.g. education, patients' ED duration and treatment type). However, we do not regard this as a limitation of our study. Rather, a heterogeneous sample increases the generalisability of findings regarding the usefulness and validity of the CASK. Furthermore, we did not obtain data allowing the calculation of a re‐test reliability of the CASK scores. This should be added in future studies.

To conclude, the German version of the CASK has proven to be a useful instrument to assess caregiver skills in parents of adolescent patients with AN and to evaluate the outcomes of skills‐based trainings based on the interpersonal cognitive maintenance model of EDs. Future studies should improve the evidence on the factor structure of the German version of the CASK and provide more detailed analyses towards its accuracy across different subgroups of caregivers.

## CONFLICT OF INTEREST

Julia Philipp, Stefanie Truttmann and Tanja Wittek conducted the SUCCEAT workshop and online interventions. No other conflict of interest is reported.

## Supporting information

Supplementary Material

Supplementary Material

Supplementary Material

Supplementary Material

Supplementary Material

## Data Availability

The data related to this manuscript are not publicly available.
