# Supplementary material for: A German version of the Caregiver Skills scale for caregivers of patients with anorexia nervosa
Source: Eur Eat Disord Rev. 2020 Dec 17;29(2):257–68. doi: 10.1002/erv.2817 (PMC7986839; doi:10.1002/erv.2817)
Supplement: Supplementary file 1 — Supplementary Material [file ERV-29-257-s004.docx]

**Fertigkeiten von Angehörigen**

(Deutschsprachige Übersetzung des Caregiver Skills (CASK) Fragebogens; Hibbs et al., 2014)

Wir interessieren uns für Ihre Gedanken als Angehörige von Patienten mit Essstörungen. Bitte seien Sie so offen und ehrlich wie möglich.

Die Aussagen beschreiben Situationen, die mit Essstörungen in Verbindung gebracht werden. Bitte geben Sie für jede dieser Aussagen an, wie überzeugt Sie sind, dass Sie in der angegebenen Art und Weise reagieren können.

**Schätzen Sie von 0 bis 100 in der untenstehenden Skala ein, wie überzeugt Sie sind, in folgender Weise zu handeln.**

| 0 | 10 | 20 | 30 | 40 | 50 | 60 | 70 | 80 | 90 | 100 |
| --- | --- | --- | --- | --- | --- | --- | --- | --- | --- | --- |

**Fast nie Gelegentlich Häufig Fast immer**

Beispiel: Eine Einschätzung von 100 bedeutet, dass Sie 100%ig überzeugt sind, dass Sie so handeln könnten, wenn Sie es möchten. Kreuzen Sie für jede Aussage die Zahl an, die Ihrer Meinung nach Ihre Überzeugung, so zu handeln am besten widerspiegelt. Sie können jede Prozentzahl von 0 bis 100 wählen (10,20, 30, usw.). Es kann sein, dass es bei manchen Fragen nicht nachvollziehbar ist, was genau gemeint ist. In diesem Fall haben Sie auch die Möglichkeit anzugeben, dass Sie die Frage nicht verstehen.

**Bitte schätzen Sie sich so ein, wie Sie sich DIESE WOCHE fühlen**, nicht wie es früher war oder wie Sie gerne sein würden. Das ist sehr wichtig. Sollten Fragen oder Aussagen nicht auf Ihre Situation zutreffen, versuchen Sie abzuschätzen, wie überzeugt Sie sind, dass Sie so handeln würden, wenn diese Situation einträfe. Die Platzhalter beziehen sich auf Ihr Kind mit einer Essstörung. Sie können hier gedanklich den Namen Ihres Kindes einsetzen.

Vielen Dank, dass Sie sich die Zeit nehmen, diesen Fragebogen auszufüllen.

**Wie überzeugt sind Sie, dass …**

1. … Sie weiterhin Dinge machen können, die Sie gerne tun, obwohl Sie sich um ___________ kümmern?

| 0 | 10 | 20 | 30 | 40 | 50 | 60 | 70 | 80 | 90 | 100 |
| --- | --- | --- | --- | --- | --- | --- | --- | --- | --- | --- |

Fast nie Gelegentlich Häufig Fast immer

| 🗆 *Ich verstehe den Sinn dieser Frage nicht.* |
| --- |

1. … Sie offen über die Essstörung und Ihre damit verbundenen Gefühle mit ___________ sprechen können?

| 0 | 10 | 20 | 30 | 40 | 50 | 60 | 70 | 80 | 90 | 100 |
| --- | --- | --- | --- | --- | --- | --- | --- | --- | --- | --- |

Fast nie Gelegentlich Häufig Fast immer

| 🗆 *Ich verstehe den Sinn dieser Frage nicht.* |
| --- |

1. … Sie offen mit *allen* direkt involvierten Familienmitgliedern über die Essstörung sprechen können?

| 0 | 10 | 20 | 30 | 40 | 50 | 60 | 70 | 80 | 90 | 100 |
| --- | --- | --- | --- | --- | --- | --- | --- | --- | --- | --- |

Fast nie Gelegentlich Häufig Fast immer

| 🗆 *Ich verstehe den Sinn dieser Frage nicht.* |
| --- |

**Wie überzeugt sind Sie, dass Sie …**

1. … Sie gegenüber ___________ verständnisvoll sein können, auch wenn Sie wegen ihr/ihm verärgert oder frustriert sind?

| 0 | 10 | 20 | 30 | 40 | 50 | 60 | 70 | 80 | 90 | 100 |
| --- | --- | --- | --- | --- | --- | --- | --- | --- | --- | --- |

Fast nie Gelegentlich Häufig Fast immer

| 🗆 *Ich verstehe den Sinn dieser Frage nicht.* |
| --- |

1. … Sie es vermeiden können, mit ___________ über die Essstörung zu streiten?

| 0 | 10 | 20 | 30 | 40 | 50 | 60 | 70 | 80 | 90 | 100 |
| --- | --- | --- | --- | --- | --- | --- | --- | --- | --- | --- |

Fast nie Gelegentlich Häufig Fast immer

| 🗆 *Ich verstehe den Sinn dieser Frage nicht.* |
| --- |

1. … Sie ruhig bleiben können, wenn Sie mit schwierigem Verhalten in Zusammenhang mit der Essstörung konfrontiert sind?

| 0 | 10 | 20 | 30 | 40 | 50 | 60 | 70 | 80 | 90 | 100 |
| --- | --- | --- | --- | --- | --- | --- | --- | --- | --- | --- |

Fast nie Gelegentlich Häufig Fast immer

| 🗆 *Ich verstehe den Sinn dieser Frage nicht.* |
| --- |

1. … Sie sich Zeit für sich selbst nehmen können, wenn Sie eine Pause brauchen?

| 0 | 10 | 20 | 30 | 40 | 50 | 60 | 70 | 80 | 90 | 100 |
| --- | --- | --- | --- | --- | --- | --- | --- | --- | --- | --- |

Fast nie Gelegentlich Häufig Fast immer

| 🗆 *Ich verstehe den Sinn dieser Frage nicht.* |
| --- |

1. … Sie mit ___________ über ihre/seine Gefühle in schwierigen und komplizierten Situationen sprechen und zuhören können?

| 0 | 10 | 20 | 30 | 40 | 50 | 60 | 70 | 80 | 90 | 100 |
| --- | --- | --- | --- | --- | --- | --- | --- | --- | --- | --- |

Fast nie Gelegentlich Häufig Fast immer

| 🗆 *Ich verstehe den Sinn dieser Frage nicht.* |
| --- |

1. … Ihnen auch sehr kleine Anzeichen einer Besserung von ___________ Mut machen?

| 0 | 10 | 20 | 30 | 40 | 50 | 60 | 70 | 80 | 90 | 100 |
| --- | --- | --- | --- | --- | --- | --- | --- | --- | --- | --- |

Fast nie Gelegentlich Häufig Fast immer

| 🗆 *Ich verstehe den Sinn dieser Frage nicht.* |
| --- |

1. … Sie weiter hoffen können, dass ___________ wieder gesund wird?

| 0 | 10 | 20 | 30 | 40 | 50 | 60 | 70 | 80 | 90 | 100 |
| --- | --- | --- | --- | --- | --- | --- | --- | --- | --- | --- |

Fast nie Gelegentlich Häufig Fast immer

| 🗆 *Ich verstehe den Sinn dieser Frage nicht.* |
| --- |

1. … Sie sich selbst zurücknehmen und darauf vertrauen können, dass ___________ die Herausforderungen des alltäglichen Lebens selbständig bewältigen kann?

| 0 | 10 | 20 | 30 | 40 | 50 | 60 | 70 | 80 | 90 | 100 |
| --- | --- | --- | --- | --- | --- | --- | --- | --- | --- | --- |

Fast nie Gelegentlich Häufig Fast immer

| 🗆 *Ich verstehe den Sinn dieser Frage nicht.* |
| --- |

**Wie überzeugt sind Sie, dass …**

1. … Sie sich gemeinsam mit ___________ auf Grenzen, Pläne oder Regeln zu Hause einigen können?

| 0 | 10 | 20 | 30 | 40 | 50 | 60 | 70 | 80 | 90 | 100 |
| --- | --- | --- | --- | --- | --- | --- | --- | --- | --- | --- |

Fast nie Gelegentlich Häufig Fast immer

| 🗆 *Ich verstehe den Sinn dieser Frage nicht.* |
| --- |

1. … Sie weiterhin auf die Einhaltung von Grenzen/Regeln bestehen und dabei einfühlsam bleiben können, auch wenn ___________ sich dagegen auflehnt?

| 0 | 10 | 20 | 30 | 40 | 50 | 60 | 70 | 80 | 90 | 100 |
| --- | --- | --- | --- | --- | --- | --- | --- | --- | --- | --- |

Fast nie Gelegentlich Häufig Fast immer

| 🗆 *Ich verstehe den Sinn dieser Frage nicht.* |
| --- |

1. … Sie es vermeiden können, Argumente gegen die Essstörung zu äußern auch wenn Sie die Argumente für logisch halten.

| 0 | 10 | 20 | 30 | 40 | 50 | 60 | 70 | 80 | 90 | 100 |
| --- | --- | --- | --- | --- | --- | --- | --- | --- | --- | --- |

Fast nie Gelegentlich Häufig Fast immer

| 🗆 *Ich verstehe den Sinn dieser Frage nicht.* |
| --- |

1. … Sie angenehme Gespräche mit ___________ führen können, die nichts mit der Essstörung zu tun haben?

| 0 | 10 | 20 | 30 | 40 | 50 | 60 | 70 | 80 | 90 | 100 |
| --- | --- | --- | --- | --- | --- | --- | --- | --- | --- | --- |

Fast nie Gelegentlich Häufig Fast immer

| 🗆 *Ich verstehe den Sinn dieser Frage nicht.* |
| --- |

1. … Sie es vermeiden können, ständig nachzufragen und das Verhalten von ___________ zu kontrollieren, auch wenn Sie sehr besorgt sind?

| 0 | 10 | 20 | 30 | 40 | 50 | 60 | 70 | 80 | 90 | 100 |
| --- | --- | --- | --- | --- | --- | --- | --- | --- | --- | --- |

Fast nie Gelegentlich Häufig Fast immer

| 🗆 *Ich verstehe den Sinn dieser Frage nicht.* |
| --- |

1. … Sie Veränderungen oder Veränderungsversuche in Bezug auf die Essstörung von ___________ loben können, auch wenn sie weniger gebracht haben, als Sie es sich erhofft haben?

| 0 | 10 | 20 | 30 | 40 | 50 | 60 | 70 | 80 | 90 | 100 |
| --- | --- | --- | --- | --- | --- | --- | --- | --- | --- | --- |

Fast nie Gelegentlich Häufig Fast immer

| 🗆 *Ich verstehe den Sinn dieser Frage nicht.* |
| --- |

1. … Sie es vermeiden können, ständig bezüglich vereinbarter Verhaltensweisen in Bezug auf die Essstörung nachzufragen oder ___________ daran zu erinnern?

| 0 | 10 | 20 | 30 | 40 | 50 | 60 | 70 | 80 | 90 | 100 |
| --- | --- | --- | --- | --- | --- | --- | --- | --- | --- | --- |

Fast nie Gelegentlich Häufig Fast immer

| 🗆 *Ich verstehe den Sinn dieser Frage nicht.* |
| --- |

**Wie überzeugt sind Sie, dass …**

1. … Sie es vermeiden können, sich mit ___________ wiederholt in Gespräche über Nahrung und Essen verstricken zu lassen?

| 0 | 10 | 20 | 30 | 40 | 50 | 60 | 70 | 80 | 90 | 100 |
| --- | --- | --- | --- | --- | --- | --- | --- | --- | --- | --- |

Fast nie Gelegentlich Häufig Fast immer

| 🗆 *Ich verstehe den Sinn dieser Frage nicht.* |
| --- |

1. … Sie das große Ganze/den Fortschritt von ___________ insgesamt im Auge behalten können?

| 0 | 10 | 20 | 30 | 40 | 50 | 60 | 70 | 80 | 90 | 100 |
| --- | --- | --- | --- | --- | --- | --- | --- | --- | --- | --- |

Fast nie Gelegentlich Häufig Fast immer

| 🗆 *Ich verstehe den Sinn dieser Frage nicht.* |
| --- |

1. … Sie es schaffen, nicht nur das Gewicht als einzigen Maßstab für den Gesundheitszustand von ___________ heranzuziehen?

| 0 | 10 | 20 | 30 | 40 | 50 | 60 | 70 | 80 | 90 | 100 |
| --- | --- | --- | --- | --- | --- | --- | --- | --- | --- | --- |

Fast nie Gelegentlich Häufig Fast immer

| 🗆 *Ich verstehe den Sinn dieser Frage nicht.* |
| --- |

1. … Sie ___________ als Person von der Krankheit (Essstörung) trennen können?

| 0 | 10 | 20 | 30 | 40 | 50 | 60 | 70 | 80 | 90 | 100 |
| --- | --- | --- | --- | --- | --- | --- | --- | --- | --- | --- |

Fast nie Gelegentlich Häufig Fast immer

| 🗆 *Ich verstehe den Sinn dieser Frage nicht.* |
| --- |

1. … Sie die Wirkung Ihres Verhaltens auf __________ reflektieren und verstehen können?

| 0 | 10 | 20 | 30 | 40 | 50 | 60 | 70 | 80 | 90 | 100 |
| --- | --- | --- | --- | --- | --- | --- | --- | --- | --- | --- |

Fast nie Gelegentlich Häufig Fast immer

| 🗆 *Ich verstehe den Sinn dieser Frage nicht.* |
| --- |

1. … Sie akzeptieren können, dass Sie nicht an der Essstörung schuld sind?

| 0 | 10 | 20 | 30 | 40 | 50 | 60 | 70 | 80 | 90 | 100 |
| --- | --- | --- | --- | --- | --- | --- | --- | --- | --- | --- |

Fast nie Gelegentlich Häufig Fast immer

| 🗆 *Ich verstehe den Sinn dieser Frage nicht.* |
| --- |

1. … Sie akzeptieren können, dass es nicht nur EINE Ursache/EINEN Auslöser für die Entstehung der Essstörung gibt?

| 0 | 10 | 20 | 30 | 40 | 50 | 60 | 70 | 80 | 90 | 100 |
| --- | --- | --- | --- | --- | --- | --- | --- | --- | --- | --- |

Fast nie Gelegentlich Häufig Fast immer

| 🗆 *Ich verstehe den Sinn dieser Frage nicht.* |
| --- |

1. … Sie Zeit mit anderen Familienmitgliedern verbringen können?

| 0 | 10 | 20 | 30 | 40 | 50 | 60 | 70 | 80 | 90 | 100 |
| --- | --- | --- | --- | --- | --- | --- | --- | --- | --- | --- |

Fast nie Gelegentlich Häufig Fast immer

| 🗆 *Ich verstehe den Sinn dieser Frage nicht.* |
| --- |

1. … Sie Ihren Angstpegel im Griff haben, so dass Sie sich nicht überwältigt fühlen?

| 0 | 10 | 20 | 30 | 40 | 50 | 60 | 70 | 80 | 90 | 100 |
| --- | --- | --- | --- | --- | --- | --- | --- | --- | --- | --- |

Fast nie Gelegentlich Häufig Fast immer

| 🗆 *Ich verstehe den Sinn dieser Frage nicht.* |
| --- |

**Auswertung** (entsprechend der englischen Original-Version, Hibbs et al., 2014)

*CASK Total Mean Score* (Score Range 0-100)

= MEAN(Item1, Item2, Item3, Item4, Item5, Item6, Item7, Item8, Item9, Item10, Item11, Item12, Item13, Item14, Item15, Item16, Item17, Item18, Item 19, Item20, Item21, Item22, Item23, Item24, Item25, Item26, Item27).

*CASK Bigger Picture Mean Score* (Score Range 0-100)

= MEAN(Item9, Item10, Item17, Item20, Item21, Item22, Item23).

*CASK Self Care Mean Score* (Score Range 0-100)

= MEAN(Item1, Item7, Item11, Item26).

*CASK Biting Tongue Mean Score* (Score Range 0-100)

= MEAN(Item16, Item18, Item19).

*CASK Insight and Acceptance Mean Score* (Score Range 0-100)

= MEAN(Item24, Item25, Item27).

*CASK Emotional Intelligence Mean Score* (Score Range 0-100)

= MEAN(Item2, Item3, Item8, Item12, Item13).

*CASK Frustration Tolerance Mean Score* (Score Range 0-100)

= MEAN(Item4, Item5, Item6, Item14, Item15).
