# Supplementary material for: A German version of the Caregiver Skills scale for caregivers of patients with anorexia nervosa
Source: Eur Eat Disord Rev. 2020 Dec 17;29(2):257–68. doi: 10.1002/erv.2817 (PMC7986839; doi:10.1002/erv.2817)
Supplement: Supplementary file 2 — Supplementary Material [file ERV-29-257-s003.docx]

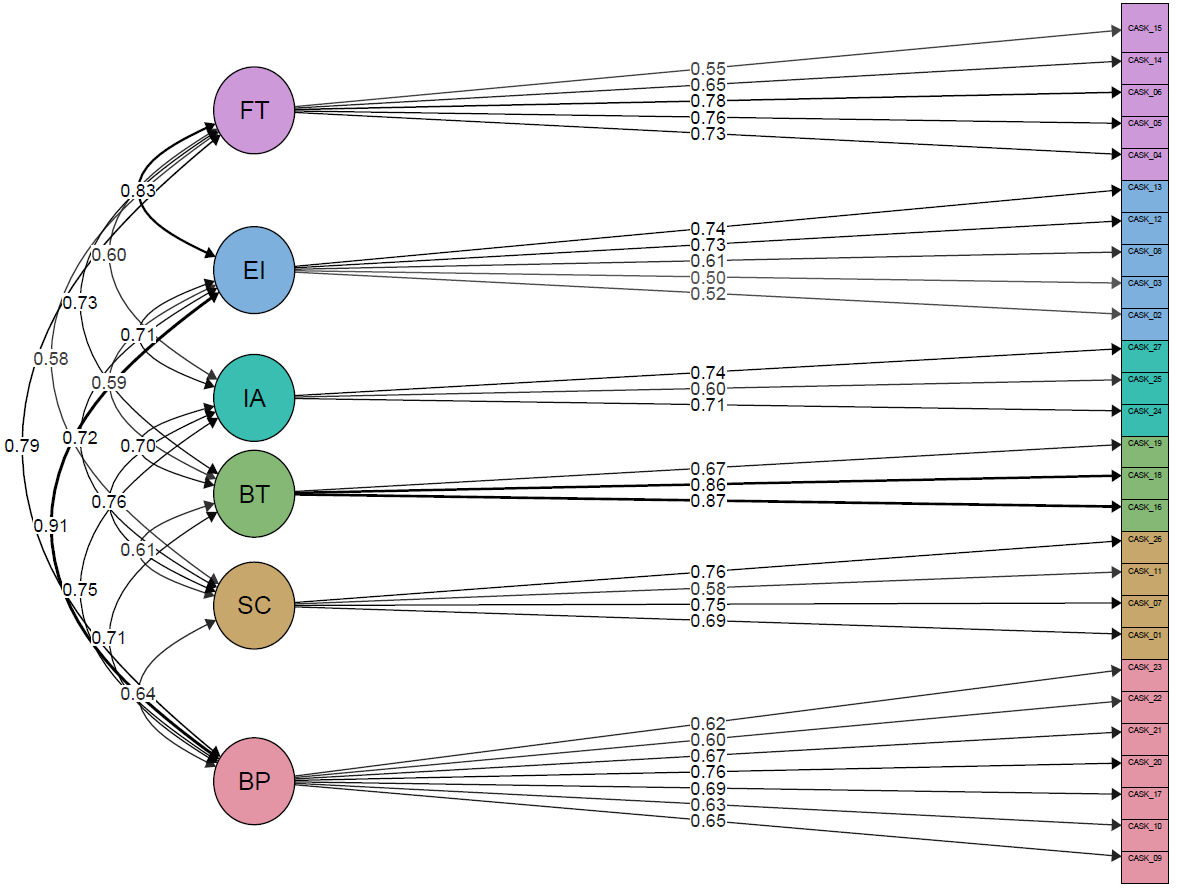


Figure S1. Path diagram of the 6-factor model of the CASK including factor loadings and correlations between latent factors
Abbreviations of the CASK factors: BG Bigger Picture, SC Self-Care, BT Biting Tongue, IA Insight & Acceptance, EI Emotional Intelligence, FT Frustration Tolerance
