# Supplementary material for: A German version of the Caregiver Skills scale for caregivers of patients with anorexia nervosa
Source: Eur Eat Disord Rev. 2020 Dec 17;29(2):257–68. doi: 10.1002/erv.2817 (PMC7986839; doi:10.1002/erv.2817)
Supplement: Supplementary file 3 — Supplementary Material [file ERV-29-257-s002.docx]

**Table S1.** Factor loadings of the re-specified CFA 6-factors model (including residual item covariances in the model)

| Factors and Items | CFA factor loadings | | | |
| --- | --- | --- | --- | --- |
|  | B | SE | *p* | Beta |
| *F1. Bigger Picture* |  |  |  |  |
| 09. Reassured by even small improv. | 12.757 | 1.561 | <.001 | .602 |
| 10. Keep hope that X will recover | 11.827 | 1.307 | <.001 | .625 |
| 17. Praise change or attempts | 14.038 | 1.088 | <.001 | .676 |
| 20. Keep your eye on X’s progress | 15.086 | 1.207 | <.001 | .767 |
| 21. Resist in relying solely on weight | 14.124 | 1.379 | <.001 | .639 |
| 22. Separate X as a person | 14.629 | 1.387 | <.001 | .595 |
| 23. Reflect and understand | 12.712 | 1.309 | <.001 | .607 |
| *F2. Self-Care* |  |  |  |  |
| 01. Keep doing things that you enjoy | 15.896 | 1.682 | <.001 | .616 |
| 07. Take some time for yourself | 17.987 | 1.593 | <.001 | .686 |
| 11. Step back and trust | 11.493 | 1.620 | <.001 | .524 |
| 26. Find time to spend with family | 17.949 | 1.238 | <.001 | .798 |
| *F3. Biting-Your-Tongue* |  |  |  |  |
| 16. Control urge enquiring checking | 21.198 | 1.264 | <.001 | .863 |
| 18. Resist constantly remind and ask | 19.865 | 1.154 | <.001 | .861 |
| 19. Avoid getting in conversations | 14.195 | 1.565 | <.001 | .605 |
| *F4. Insight and Acceptance* |  |  |  |  |
| 24. Accept that ED is not your fault | 18.704 | 2.028 | <.001 | .687 |
| 25. Insight there is no one cause | 12.368 | 1.855 | <.001 | .592 |
| 27. Manage your anxiety levels | 17.544 | 1.616 | <.001 | .735 |
| *F5. Emotional Intelligence* |  |  |  |  |
| 02. Discuss and explain feelings | 11.729 | 2.177 | <.001 | .416 |
| 03. Discuss the ED openly with family | 12.082 | 1.983 | <.001 | .479 |
| 08. Talk and listen with X emotions | 11.415 | 2.123 | <.001 | .453 |
| 12. Agree boundaries-plans | 17.111 | 1.467 | <.001 | .774 |
| 13. Uphold boundaries / rules | 16.628 | 1.320 | <.001 | .793 |
| *F6. Frustration Tolerance* |  |  |  |  |
| 04. Be understanding towards X | 13.272 | 1.206 | <.001 | .711 |
| 05. Avoid drawn into arguments | 16.137 | 1.094 | <.001 | .741 |
| 06. Be calm with difficult ED behavior | 17.111 | 1.068 | <.001 | .801 |
| 14. Control the urge to argue | 14.712 | 1.494 | <.001 | .634 |
| 15. Pleasant verbal interactions | 11.223 | 1.429 | <.001 | .555 |

Note: Residual item covariances of items #1, #2, #3, #10, #11, #15, #19 with other items specified in the CFA model; Model fit: RMSEA = .051; SRMR = .063; CFI = .937
