# Supplementary material for: A German version of the Caregiver Skills scale for caregivers of patients with anorexia nervosa
Source: Eur Eat Disord Rev. 2020 Dec 17;29(2):257–68. doi: 10.1002/erv.2817 (PMC7986839; doi:10.1002/erv.2817)
Supplement: Supplementary file 4 — Supplementary Material [file ERV-29-257-s001.docx]

**Table S2.** Internal consistencies (Cronbach α) of CASK scales by caregiver’s sex

|  | **Cronbach α** | |  | **Feldt Test** | |
| --- | --- | --- | --- | --- | --- |
| CASK scales | **Mothers** | **Fathers** |  | **W** | ***p*** |
| CASK Total | .93 | .95 |  | 0.714 | .112 |
| CASK Bigger Picture | .83 | .89 |  | 0.647 | .042 |
| CASK Self-Care | .78 | .77 |  | 0.957 | .405 |
| CASK Biting Tongue | .85 | .74 |  | 0.577 | **.004** |
| CASK Insight and Acceptance | .71 | .66 |  | 0.853 | .224 |
| CASK Emotional Intelligence | .76 | .84 |  | 0.667 | .049 |
| CASK Frustration Tolerance | .82 | .81 |  | 0.947 | .391 |

Note: Bonferroni-adjusted significance level (α = .006) used
