# Supplementary material for: A German version of the Caregiver Skills scale for caregivers of patients with anorexia nervosa
Source: Eur Eat Disord Rev. 2020 Dec 17;29(2):257–68. doi: 10.1002/erv.2817 (PMC7986839; doi:10.1002/erv.2817)
Supplement: Supplementary file 5 — Supplementary Material [file ERV-29-257-s005.docx]

**Table S3a.** CASK norm values for the total sample (N = 233 male and female caregivers of adolescents patients with anorexia nervosa)

| Percentile | CASK Raw Mean Scores | | | | | | |
| --- | --- | --- | --- | --- | --- | --- | --- |
|  | Total | Bigger Picture | Self-Care | Biting-Your-Tongue | Insight and Acceptance | Emotional Intelligence | Frustration Tolerance |
| 1% | ≤30.23 | ≤27.39 | ≤20.83 | ≤16.67 | ≤20.00 | ≤24.64 | ≤22.66 |
| 5% | 43.24 | 45.71 | 29.13 | 20.00 | 35.00 | 37.20 | 40.00 |
| 10% | 49.31 | 54.29 | 37.50 | 30.00 | 43.33 | 44.00 | 46.60 |
| 15% | 53.20 | 58.49 | 40.00 | 33.33 | 50.00 | 50.00 | 50.00 |
| 20% | 56.00 | 61.43 | 43.00 | 40.00 | 53.33 | 54.00 | 52.00 |
| 25% | 58.15 | 62.86 | 45.63 | 46.67 | 60.00 | 56.00 | 56.00 |
| 30% | 60.00 | 65.71 | 50.00 | 46.67 | 63.33 | 58.00 | 59.80 |
| 35% | 62.50 | 68.57 | 52.50 | 50.00 | 66.67 | 60.40 | 62.00 |
| 40% | 64.81 | 71.43 | 55.00 | 53.33 | 70.00 | 64.00 | 64.00 |
| 45% | 66.91 | 72.86 | 57.50 | 56.67 | 73.33 | 68.00 | 67.93 |
| 50% | 68.50 | 75.00 | 62.50 | 60.00 | 76.67 | 70.00 | 70.00 |
| 55% | 70.37 | 77.21 | 65.00 | 63.33 | 76.67 | 73.60 | 72.00 |
| 60% | 72.65 | 80.00 | 67.50 | 66.67 | 80.00 | 76.00 | 74.00 |
| 65% | 74.10 | 81.46 | 70.00 | 70.00 | 83.33 | 78.00 | 77.73 |
| 70% | 76.93 | 84.29 | 72.50 | 73.33 | 83.33 | 82.00 | 78.00 |
| 75% | 79.35 | 85.71 | 75.00 | 76.67 | 86.67 | 84.00 | 80.00 |
| 80% | 81.11 | 87.14 | 77.50 | 80.00 | 90.00 | 86.00 | 82.00 |
| 85% | 83.75 | 90.00 | 82.50 | 83.33 | 93.33 | 88.00 | 84.05 |
| 90% | 86.30 | 92.86 | 87.50 | 86.67 | 96.67 | 92.00 | 88.00 |
| 95% | 89.63 | 96.14 | 92.50 | 90.00 | 98.33 | 96.80 | 92.00 |
| 99% | ≥96.92 | ≥99.56 | 100.00 | 100.00 | 100.00 | 100.00 | 100.00 |

**Table S3b.** CASK norm values for female caregivers (N = 177 mothers) of adolescents patients with anorexia nervosa

| Percentile | CASK Raw Mean Scores | | | | | | |
| --- | --- | --- | --- | --- | --- | --- | --- |
|  | Total | Bigger Picture | Self-Care | Biting-Your-Tongue | Insight and Acceptance | Emotional Intelligence | Frustration Tolerance |
| 1% | ≤30.57 | ≤31.99 | ≤18.85 | ≤16.67 | ≤17.70 | ≤25.54 | ≤23.56 |
| 5% | 43.39 | 46.93 | 27.38 | 20.00 | 30.00 | 37.70 | 38.00 |
| 10% | 48.06 | 54.29 | 36.75 | 26.67 | 40.00 | 44.00 | 46.00 |
| 15% | 51.11 | 57.80 | 40.00 | 30.00 | 46.67 | 49.10 | 49.40 |
| 20% | 55.19 | 61.43 | 42.83 | 36.67 | 51.33 | 54.00 | 52.00 |
| 25% | 57.62 | 62.86 | 45.00 | 41.67 | 53.33 | 56.00 | 54.50 |
| 30% | 58.56 | 64.29 | 50.00 | 46.67 | 60.00 | 58.20 | 58.00 |
| 35% | 60.35 | 68.27 | 50.00 | 50.00 | 60.00 | 60.00 | 60.00 |
| 40% | 62.89 | 70.00 | 52.50 | 50.67 | 63.33 | 64.00 | 62.00 |
| 45% | 65.06 | 72.86 | 55.00 | 53.50 | 68.83 | 68.00 | 64.20 |
| 50% | 67.12 | 74.29 | 58.75 | 56.67 | 70.00 | 70.00 | 68.00 |
| 55% | 69.37 | 77.14 | 62.50 | 60.00 | 73.33 | 72.00 | 70.00 |
| 60% | 70.38 | 78.86 | 65.00 | 66.00 | 76.67 | 74.00 | 72.00 |
| 65% | 73.09 | 80.75 | 67.50 | 66.67 | 80.00 | 76.10 | 74.00 |
| 70% | 74.07 | 83.93 | 70.00 | 70.00 | 80.00 | 80.00 | 77.80 |
| 75% | 77.04 | 85.71 | 72.50 | 73.33 | 83.33 | 82.00 | 80.00 |
| 80% | 79.85 | 87.14 | 76.50 | 76.67 | 85.00 | 84.00 | 82.00 |
| 85% | 83.50 | 90.00 | 81.13 | 80.00 | 90.00 | 86.90 | 84.00 |
| 90% | 86.25 | 92.02 | 85.00 | 84.00 | 93.33 | 92.00 | 88.00 |
| 95% | 89.31 | 94.39 | 92.50 | 93.33 | 96.67 | 94.30 | 92.00 |
| 99% | ≥96.29 | 100.00 | 98.08 | 100.00 | 100.00 | 100.00 | 100.00 |

**Table S3c.** CASK norm values for male caregivers (N = 56 fathers) of adolescents patients with anorexia nervosa

| Percentile | CASK Raw Mean Scores | | | | | | |
| --- | --- | --- | --- | --- | --- | --- | --- |
|  | Total | Bigger Picture | Self-Care | Biting-Your-Tongue | Insight and Acceptance | Emotional Intelligence | Frustration Tolerance |
| 1% | ≤21.85 | ≤17.14 | ≤32.50 | ≤26.67 | ≤40.00 | ≤4.00 | ≤22.00 |
| 5% | 39.88 | 42.50 | 35.00 | 30.00 | 45.67 | 35.60 | 42.00 |
| 10% | 54.63 | 50.71 | 37.50 | 36.67 | 73.33 | 42.30 | 47.20 |
| 15% | 57.28 | 58.57 | 40.00 | 42.67 | 73.33 | 50.00 | 52.00 |
| 20% | 61.48 | 62.86 | 43.50 | 47.33 | 73.33 | 52.80 | 54.80 |
| 25% | 64.63 | 66.79 | 50.63 | 53.33 | 76.67 | 55.00 | 60.00 |
| 30% | 66.30 | 70.00 | 57.50 | 56.67 | 76.67 | 58.00 | 62.40 |
| 35% | 67.46 | 71.67 | 60.00 | 60.00 | 79.67 | 64.00 | 67.60 |
| 40% | 70.37 | 72.86 | 63.17 | 60.00 | 83.33 | 67.60 | 74.00 |
| 45% | 72.30 | 75.36 | 65.00 | 63.33 | 86.67 | 72.40 | 74.40 |
| 50% | 75.00 | 77.86 | 71.25 | 66.67 | 86.67 | 78.00 | 78.00 |
| 55% | 76.85 | 80.00 | 72.79 | 70.00 | 86.67 | 78.00 | 78.00 |
| 60% | 79.26 | 81.43 | 75.00 | 73.33 | 86.67 | 80.00 | 78.00 |
| 65% | 80.00 | 84.29 | 75.13 | 76.67 | 90.00 | 82.00 | 80.00 |
| 70% | 80.37 | 84.29 | 77.50 | 80.00 | 93.33 | 84.00 | 80.00 |
| 75% | 81.20 | 85.71 | 79.38 | 83.33 | 95.00 | 86.00 | 82.00 |
| 80% | 81.85 | 86.67 | 85.00 | 83.33 | 96.67 | 86.00 | 84.00 |
| 85% | 85.97 | 93.93 | 87.50 | 86.67 | 96.67 | 88.00 | 88.00 |
| 90% | 88.52 | 96.07 | 90.75 | 86.67 | 98.67 | 96.80 | 88.00 |
| 95% | 91.57 | 97.50 | 100.00 | 90.00 | 100.00 | 100.00 | 92.80 |
| 99% | ≥ 92.00 | ≥ 98.00 | 100.00 | ≥ 91.00 | 100.00 | 100.00 | ≥ 93.00 |
